# Single-Day Simulation-Based Training Improves Communication and Psychiatric Skills of Medical Students

**DOI:** 10.3389/fpsyt.2020.00221

**Published:** 2020-03-20

**Authors:** Doron Amsalem, Doron Gothelf, Omer Soul, Alexandra Dorman, Amitai Ziv, Raz Gross

**Affiliations:** ^1^The Child Psychiatry Division, The Edmond and Lily Safra Children’s Hospital, Sheba Medical Center, Tel Hashomer, Israel; ^2^Sackler Faculty of Medicine, Tel Aviv University, Tel Aviv, Israel; ^3^MSR–Israel Center for Medical Simulation, Sheba Medical Center, Tel Hashomer, Israel; ^4^Sackler Faculty of Medicine and Sagol School of Neuroscience, Tel Aviv University, Tel Aviv, Israel; ^5^Sheba Medical Center, Tel Hashomer, Israel; ^6^Division of Psychiatry, Sheba Medical Center, Tel Hashomer, Israel

**Keywords:** simulation, psychiatry rotation, medical education, medical students, stigma

## Abstract

**Background:**

Use of standardized (or simulated) patients (SP) is considered an effective teaching method for improving clinical and communication skills. This study assesses the effect of a single-day simulated patients (SP)-based training course on medical students’ communication and basic skills in clinical psychiatry during their psychiatry rotation in a university-affiliated tertiary medical center.

**Methods:**

Forty-two third-year medical students participated. Communication and basic skills in clinical psychiatry were evaluated by a modified Four Habits Coding Scale (4HCS) and the psychiatric interview coding scale before and after SP training. An actual patient interview by the students 1 week after the training was evaluated by an attending psychiatrist blinded to the student’s score during the SP-based training. Self-report questionnaires on satisfaction from the training and its impact on their self-confidence were administered at the end of training.

**Findings:**

The mean pre-training 4HCS score of 33.9 increased to 52.3 post-training (*p* < 0.001). The mean psychiatric interview coding scale score increased from 4.33 to 5.36 (*p* = 0.002). The self-report questionnaire yielded a mean score of 4.21 on a 1–5 Likert scale, implying high levels of satisfaction and self-confidence.

**Conclusions:**

A single SP-based training course of medical students sufficed to improve clinical and communication skills in psychiatric settings and enhance their subjective perception of those skills.

## Introduction

Simulation has a unique role as an effective training method in bridging the gap between education and clinical practice. The use of trained actors as simulated patients (SP) offers numerous advantages in medical education that have been well reviewed in the literature (1, 2). Practicing pre-speciﬁed and predictable clinical scenarios in a completely safe environment bears no risk of harm to patients by inexperienced students or residents, and allows the educator and trainee to focus their attention on the aspect of patient-doctor interactions most relevant to the specific training needs (3, 4).

Although SP-based methodology has a rich history in medical education, its use in psychiatry training has started to emerge only more recently (5, 6). Simulations in psychiatry focus on promoting better interviewing, communication, and diagnostic skills, and the understanding of the underlying personal human narrative of patients. There are several applications for SP in psychiatry: 1. To enhance exposure of a broader range of patients and psychopathology (7); 2. To evaluate the clinical skills of students and residents by means of a structured exam, such as the Objective Structured Clinical Examinations (8–11); 3. To practice psychotherapeutic techniques (12); 4. Stigma reduction among medical students (13). An important advantage of SP-based training is that it is free of the potential ethical and safety issues that might arise when teaching is done with real patients (3, 4). There are, however, also several limitations to SPs (14). Accurately simulating clinical encounters may be difficult, as simulated scenarios often tend to be “textbook cases” that might not represent the complexity of real patients (15).

Data on the implementation of SP-based training of medical students during rotation in psychiatry are sparse. One study described a course developed to help students gain broader clinical experience (16),but evaluation was based only on the subjective report by the students, without any objective evaluation. Another study compared scores of the psychiatric component of the clinical competency examination at the end of the fourth year between medical students who were trained using SPs during their third year and those who were trained as usual (17). A limitation of that study is that it did not assess the effect of SP-based training on students’ performance with real patients. Another study has shown good validity of SP-based evaluation of students’ communication skills (18). Therefore, the goal of our study was to assess the effect of a single-day SP-based training course on medical students’ communication and basic skills in clinical psychiatry, and to compare between evaluations of students’ performance in the simulation lab vs. clinical setting.

## Materials and Methods

### Design and Participants

This study was designed to evaluate communication and psychiatric skills of medical students before and after a single, simulation-based training. Participants included 3^rd^ year medical students from two affiliated universities, during their 6 weeks rotation in psychiatry in a large general hospital in the center of Israel: St. George University of London (SGUL) program (n = 30), and Tel-Aviv University (TAU) (n = 14). One student from each university declined participation in the study. The SGUL program study was conducted in January 2018, and the TAU program study was conducted in May 2018.

### Procedure

**Figure 1:**
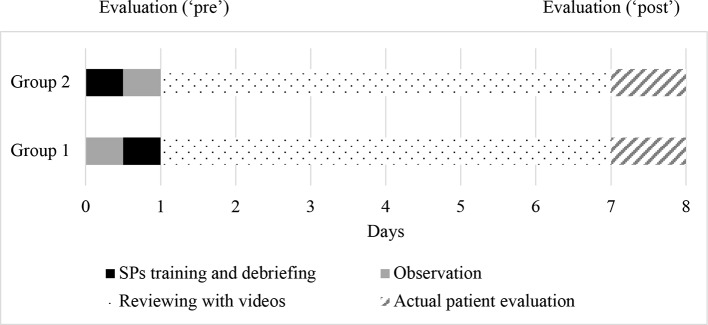
Time Chart.

The study included a one-day training with SPs, at the Israel Center for Medical Simulation (MSR), followed by a patient interview in a psychiatric ward 1 week later. The 1-day training with SPs consisted of two scenarios, each followed by small group debriefing discussion sessions. In each of the training days, students were randomly divided into two groups, with up to seven participants per group. Microphones and one-way mirrors enabled to the students who were trained second to observe the SP encounter of the students who were trained first (see **Figure 1**). Video cameras recorded the encounters for further analysis, feedback, and reflection during debriefing sessions with psychiatrists. Each student encountered one SP and observed at least two other encounters.

Following the 1-day training with SPs, students received their full-length video and a list of questions as a take-home assignment (e.g., “did I use open or close ended questions?”). One week later, the students interviewed real patient in ward settings, and were observed by a different psychiatrist. The video-based debriefing and patients’ interviews were led by psychiatrists who are experts in the art of video-based debriefing, who highlighted different aspects of communication skills and core elements of the mental status examination.

The SP-based scenarios included psychiatric vignettes of two patients. The first vignette describes a patient with a diagnosis of major depressive disorder and drug abuse, admitted to a close-ward (locked unit) due to recent suicide attempt. The second vignette describes an outpatient with posttraumatic stress disorder who suffers chronic pain and refuses to use medications. The vignettes included all components of a comprehensive psychiatric interview, i.e., identifying information, chief complaint, history of present illness, past psychiatric and medical histories, family and social history, and history of substance use and dependence. The duration of each scenario was 15 min. The project was approved by the Sheba Medical Center Institutional Review Board, and all participants signed an informed consent form.

### Evaluation

We conducted real-time evaluations of both SP and real patient interviews. The Four Habits Coding Scheme (4HCS) (19) and a Mental Status Coding Scale (MSCS) were used to assess students during the training and patient interviews. The 4HCS includes 23 items in four different habits domains. Habit 1: Invest in the beginning; habit 2: Elicit patients’ perspective; habit 3: Demonstrate empathy; habit 4: Invest in the end. Since habit 4 refers to future treatment plan, and thus is irrelevant in the context of the study, we removed this domain from our coding scheme. Similarly, the first item of habit 1 (“show familiarity”) was omitted, since the interview was the students’ first encounter with the SP. Overall, we used 12 items of the 4HCS.

The 4HCS was translated into Hebrew and then back-translated by bilingual authors (Doron Amsalem and Alexandra Dorman). Discrepancies in translation were discussed and corrected by the translators and a third author (Doron Gothelf). Next, inter-rater reliability of the Hebrew version of the 4HCS between the experts was tested. A total of three videos of psychiatric interviews conducted during the previous school year by medical students from TAU were evaluated independently using the 4HCS. Following each evaluation, scores were compared and differences in scoring discussed. We computed Kappa coefficient for each evaluation separately, and observed an improvement in inter-rater reliability, from K=0.53 to 0.83.

The MSCS was developed by the authors to assess clinical skills. The MSCS consists of six items representing core features of the mental status exam (suicidality, mood, affect, thought process, delusions, and hallucinations) and is aimed at evaluating the student’s performance following the SP training. The students’ MSCS scores were compared with t scores determined by two board-certified, experienced psychiatrists, concurrent with the students’ interviews, and considered as “gold standard”. At the end of the training participants completed also a 5-point Likert-scale questionnaire on perceived knowledge, competence, and comfort in recognizing psychiatric symptoms in clinical settings.

### Statistical Analysis

Mean scores and standard deviations were calculated for each of the three 4HCS domains used in the study. We calculated the percentage of students who were correct on each of the six core features of the MSCS before and after the training. To produce a total score for the MSCS, each student was assigned a score of 0 or 1 per feature (1=correct answer, 0=incorrect or not exist; range=0–6). We used individual scores to compute before- and after training mean group scores and standard deviations. McNemar test was used to compare proportions and paired t-tests to compare mean scores. A two-tailed p-value of 0.05 was determined as the threshold of significance. Statistical analyses were done using IBM SPSS software, version 25.0.

## Results

Forty-two of the 44 students we approached (95%) have agreed to participate in the study. Nineteen participants (45%) were female, 4 (10%) were aged of 23–26 years, 21 (50%) were 27–29 years, and 17 (40%) were 30 years or older (**Table 1**). The 4 HCS scores were not significantly different between male and female participants, both before- and after training (p=0.57, p=0.51, respectively), and between SGUL and TAU (p=0.88, p=0.67 respectively).

**Table 1:** Demographic data.

Variable		n (%)
Sex		
	Male	23 (55)
	Female	19 (45)
Age, years		
	23–26	4 (10)
	27–29	21 (50)
	≥30	17 (40)
Program		
	SGUL	29 (69)
	Tel Aviv University	13 (31)

SGUL, St. George University of London.

Students’ scores in all three domains of the adapted 4-HCS, as well as the total scores, were significantly improved following training (**Table 2**). Students were randomly divided into two groups, with each group observing the other during the simulated interviews. Students in the group training second had the opportunity to observe the first group *prior* to their own training. The second group had significantly higher pre- training score compared to the first group (39.1 ± 6.0 vs. 29.2 ± 5.8, *t* = −5.4, *p <*0.001), but there was no difference in mean post- training scores (52.4 ± 6.4 vs. 52.3 ± 7.5, *t* = −0.03, *p* =0.97). Mean scores increased significantly in both groups: 29.2 ± 5.8 to 52.3 ± 7.5 (*t* = −11.2, *df* = 21, *p <*0.001) for the first group, and 39.1 ± 6.0 to 52.4 ± 6.4 (*t* = −6.9, *df* = 19, *p <*0.001) for the second group.

**Table 2:** The Four Habits Coding Scale^a^ scores before and after training (n = 42)^b^.

Habit	Pre-training Mean (SD)	Post-training Mean (SD)	t^*^	*df*	*p* value
1: Invest in the beginning	13.7 (2.9)	21.9 (3.1)	-11.6	41	**< .001**
2: Elicit patient’s perspective	8.3 (2.7)	13.2 (2.1)	-8.9	41	**< .001**
3: Demonstrate empathy	11.9 (3.3)	17.3 (2.7)	-8.8	41	**< .001**
Total	33.9 (7.7)	52.3 (6.9)	-11.5	41	**< .001**

^a^Habit 4 (invest in the end) is not relevant.

Scores ranged from 5–25 (habit 1); 3–15 (habit 2); 4–20 (habit 3); 12–60 (total). A higher score indicates better performance.

*Paired t-test.

The significance of bolded texts is P < .001

^b^p<0.001

Total mean scores for the MSCS were also significantly improved following the training (**Table 3**). The proportion of students who estimated core clinical features correctly for all items has increased after training. Noticeably, ability to identify correctly impaired thought process, a common pitfall among students, has improved from 60% to 98%.

**Table 3:** Students’ Scores on the MSCS before and after training (*n* = 42)^a^.

Signs and Symptoms	Pre-training	Post-training	p^*^		
	*n* (%)	*n* (%)			
Suicidality	26 (62)	34 (81)	0.096		
Mood symptoms	35 (83)	36 (86)	0.554		
Impaired affect	30 (71)	35 (83)	0.332		
Presence of delusions	32 (76)	38 (90)	0.146		
Presence of hallucinations	34 (81)	40 (95)	0.109		
Impaired thought process	25 (60)	41 (98)	**< .001**		
	Mean (SD)	Mean (SD)	t^**^	df^***^	*p*
MSCS total score	4.33 (1.58)	5.36 (0.95)	3.33	41	**0.002**

^a^Scores represent summary of correct (1 point) and incorrect (0 points) items. Range = 0–6.

*McNemar’s test.

**t-test.

***Degrees of freedom.

MSCS, Mental Status Coding Scale.

The significance of bolded texts is P = .002

Mean score of the 9-items self-report questionnaire filled at the end of the training day was 4.21 ± 0.98 (range=1–5), implicating high satisfaction and self-confidence. The items with the highest score were: “To what extent do you think you will use the tools you purchased during this day” (4.56 ± 0.81); “To what extent did you feel that the encounter with the actors is an important learning experience for acquiring communication skills in dealing with people in daily life?” (4.51 ± 0.84); “To what extent has the discussion with the video contributed to learning?” (4.39 ± 1.02); and “To what extent was the discussion fruitful and productive?” (4.51 ± 0.81).

## Discussion

This study aimed to evaluate whether a single-day SP-based training during psychiatry rotation is effective in improving communication and psychiatric skills of medical students in real-life clinical setting. Overall, we found a significant improvement in all the 4HCS domains (investing in the beginning, elicit patient’s perspective, and demonstrate empathy), and in the mean MSCS score, implying improvement in both communication and clinical skills. More specifically, we found a statistically significant improvement in identification of impaired thought process, and a statistical trend for improvement in suicide risk assessment. Scores of the self-report questionnaire indicated high satisfaction and self-confidence of the students following the training.

There are several strengths to our study. First, the very high response rate (95%) practically eliminated the possibility of selection bias. Second, blinding of the experts that conducted the post-training evaluations to the pre-training scores ensured that evaluations were not be biased by an *a priori* impression of the student. Third, actors were trained in person by psychiatrists involved in daily clinical practice, and the scripts for the SP-based training were prepared and reviewed in detail by those psychiatrists (RG and DG), making the SP-based training as close to real life clinical setting as possible.

An important advantage of our study over a previous study with similar findings (17), is that we only used SPs for pre-training evaluations of communication skills, while post training evaluations were done with real patients in ward setting. To the best of our knowledge, there are no published studies that examined the efficacy of SPs in improving communication skills with real patients. Our findings demonstrate the pertinence of SP-based training for clinical settings and support its utility in psychiatry rotation curriculum.

Another advantage of our study is that while previous studies have used subjective self-report, we used objective evaluations by experts to assess the improvement in clinical skills. For example, a study (16) that reported positive results with SP-based training of 3^rd^ year medical students to interview patients with psychiatric disorders and with early dementia. Another study (20) that used SPs to teach mental status examination found that students reported increased comfort and perceived competence in their clinical skills, and performed better on knowledge-based test. Statistically significant result has been obtained only in one domain most likely as a result of lack of statistical power due to relatively small sample size.

There are also several limitations to our study. First, our sample consisted of only 42 students from two classes of two medical schools, thus limiting generalizability and statistical power. Second, the modified 4HCS was not validated for this study. Third, we used SPs for “before” and real patients for “after”, and the observed improvement in skills might possibly represent, at least partially, different patterns of interaction rather than shear improvement in skills, in addition, the study lacked long-term follow-up outcomes. Forth, our study design used of before- and after comparisons for the same participant, lacking a non-training control group or comparison to other teaching methods, such as role play or video-based lecture. Consequently, improvement in communication and clinical skills might be attributed in part to other rotation-related factors. Furthermore, during the week between training and the follow-up evaluation, students continued their rotation and had other psychiatric learning experiences. Hence, some of the observed improvement in skills might plausibly be attributed to that additional training. Finally, using a metric to capture the feedback from the SP might make it more informative and useful.

## Conclusions

In conclusion, using SPs in psychiatric training may help medical students gain broader experience in clinical psychiatric assessment and diagnosis, enhance communication skills, build empathy, and possibly reduce stigma towards psychiatry. Simulated training module routinely included in the clerkship training experience might also give medical students the opportunity to view the work in the field from closer quarters. Our study showed significant results in improving both clinical and communication skills with the use of SPs. The main novelty of our study is that we used real patients and objective evaluation by experts to assess the students’ performance following SP training. Further research is needed to examine the long-term effects of comprehensive simulation-based teaching methods on interviewing, communication, and diagnostic skills of medical students.

### Clinical Implications

A single SP-based training course of medical students sufficed to improve clinical and communication skills in psychiatric settings and enhance their subjective perception of those skills.

## Data Availability Statement

All datasets generated for this study are included in the article/supplementary material.

## Ethics Statement

The project was approved by the Sheba Medical Center Institutional Review Board. The patients/participants provided their written informed consent to participate in this study.

## Author Contributions

DA, DG, AD, OS, and AZ developed the theoretical formalism, performed the analytic calculations, and performed the numerical simulations. All authors contributed to the final version of the manuscript. RG supervised the project.

## Funding

The study was supported by the MSR.

## Conflict of Interest

The authors declare that the research was conducted in the absence of any commercial or financial relationships that could be construed as a potential conflict of interest.
